# Beyond structure: spectroscopic imaging in cryogenic electron microscopy

**DOI:** 10.1016/j.sbi.2026.103304

**Published:** 2026-06-16

**Authors:** Jongbeom Kim, Eric A. Stach, Yi-Wei Chang

**Affiliations:** 1 Department of Materials Science and Engineering, School of Engineering and Applied Science, University of Pennsylvania, Philadelphia, PA, 19104, USA; 2 Department of Biochemistry and Biophysics, Perelman School of Medicine, University of Pennsylvania, Philadelphia, PA, 19104, USA; 3 Institute of Structural Biology, Perelman School of Medicine, University of Pennsylvania, Philadelphia, PA, 19104, USA

## Abstract

Cryogenic electron microscopy (cryo-EM) has transformed structural biology by enabling near-atomic resolution imaging of macromolecules and the direct visualization of molecular architectures in native cellular environments. However, conventional cryo-EM, although outstanding at elucidating molecular density and morphology, provides little direct information about chemical composition or molecular state. Here, we review recent advances in scanning-based and spectroscopic electron microscopy that extend cryo-EM beyond phase contrast, including Z-contrast imaging, energy-dispersive X-ray spectroscopy, and electron energy-loss spectroscopy. We highlight how these techniques enable label-free mapping of elemental distributions and chemical states in beam-sensitive biological specimens, and we discuss emerging workflows that integrate spectroscopy with cryogenic sample preparation and correlative imaging. Together, these developments position spectroscopic cryo-EM as a powerful approach for linking structure, composition, and function across molecular and cellular scales.

## Structural resolution achieved, but chemical context lacking

Over the past decade, cryogenic electron microscopy has dramatically expanded in both attainable resolution and biological scope. Advances in instrumentation, direct electron detectors, and image processing algorithms have pushed single-particle cryo-EM to near-atomic resolution [1,2] even for proteins once considered too small, with structures of 36—55 kDa now resolved at ~2 Å [3,4]. These milestones underscore that cryo-EM has matured into a broadly applicable, high-resolution structural technique. In parallel, cryo-electron tomography (cryo-ET) has extended structural analysis beyond purified molecules into native cells and tissues. Improvements in vitrification [5], focused ion beam (FIB) milling [6-9], and data analysis [10-15] now enable detailed resolution of macromolecular complexes directly inside intact cells, organelles, and even multicellular specimens.

Despite these advances, both single-particle and tomographic cryo-EM share a fundamental limitation: image contrast is dominated by phase contrast, which results from electrons that are scattered by the specimen’s electrostatic potential. As a result, cryo-EM can resolve molecular density and morphology but offers little direct information about chemical composition or molecular state. Dense features may arise from proteins, lipids, metals, or mineralized phases yet remain indistinguishable in phase-contrast images.

As biological functions are not only governed by molecular structural arrangements but also by complex interactions and the local distribution of chemical components, this lack of chemical context in cryo-EM images limits its ability to answer certain scientific questions. For example, recent work showing microbial bioaccumulation of environmental toxins (e.g., per- and polyfluoroalkyl substances) highlights the need to localize specific elements during this cellular process to understand the mechanism [16]. Similarly, biomolecular condensates formed by liquid—liquid phase separation are exquisitely sensitive to local ionic environments, a property not discernible from morphology alone [17]. In this way, spectroscopy can be used to determine how spatial heterogeneity within condensates reflects underlying differences in local composition, such as ion partitioning or relative enrichment of specific biomolecular components. Such spectral information could provide insight into how condensate structure relates to local chemistry in ways that are not accessible by structural imaging alone. Fluorescent chemical probe labeling strategies may help address some of these challenges. In particular, emerging cryo-fluorescence lifetime imaging approaches offer molecularly specific information beyond fluorescence intensity and emission spectra [18]. However, such labeling can perturb native structure. Furthermore, the inherent resolution limit of optical microscopy is frequently insufficient for precisely correlating features required for electron microscopy analysis [19]. These considerations motivate the development of alternative contrast mechanisms to directly access chemical composition without exogenous labels.

This review examines the emerging integration of spectroscopic methods with cryo-EM to access chemical composition alongside structure. We discuss recent progress in cryo-EELS and cryo-EDS, assess current capabilities and limitations, and consider how compositional mapping may expand the scope of structural biology.

## Scanning electron imaging and the emergence of Z-contrast

In addition to broad beam electron illumination, imaging can also be performed in scanning mode. This approach is implemented in two principal modalities: scanning electron microscopy (SEM), in which signals are collected from the specimen surface, and scanning transmission electron microscopy (STEM), where transmitted electrons are detected as the probe traverses the specimen. In both cases, a focused probe rasters across the specimen while detectors record emitted (SEM) and transmitted (STEM) electrons at each position. This scanning paradigm expands the available contrast mechanisms beyond phase contrast. It enables selective detection of elastically and inelastically scattered electrons, effectively decoupling image formation from mass density alone.

Recent studies have further demonstrated that vitrified cells and tissues can be effectively imaged by cryo-SEM using low-kV secondary electron detection [20,21]. In this regime, contrast primarily reflects surface potential and charging variations across the specimen surface, enabling visualization of light-element-rich biological material in an unstained, near-native state. Integration of SEM imaging with focused ion beam (FIB) milling has additionally enabled mill-and-view volume imaging, allowing nanoscale 3D reconstruction of cellular ultrastructure [22-25]. When implemented under cryogenic conditions, cryo-FIB-SEM operates directly on vitrified samples and supports multiscale correlative workflows in which volume imaging maps cellular context and guides the preparation of thin lamellae for subsequent cryo-ET analysis [26].

An important outcome of scanning electron imaging is atomic number (Z) contrast, which arises from elastic Rutherford scattering and increases strongly with atomic number. Because biological specimens are largely amorphous, diffraction-based contrast effects that complicate crystalline samples are minimal. As a result, scattering intensity can be interpreted primarily in terms of composition rather than crystal orientation or channeling [27]. In SEM, Z-contrast is typically detected via backscattered electrons and has long been exploited in biological imaging using heavy-metal stains.

While secondary electron imaging provides reasonable contrast for light elements such as C, H, O and N, the primary constituents of biological specimens, heavier elements like P, S, Ca and Fe are often naturally enriched in specific cellular compartments. These elements generate enhanced scattering signals and can therefore be effectively distinguished using Z-contrast in backscattered electron imaging. For example, cryo-FIB-SEM Z-contrast imaging has distinguished mineralized cartilage and bone trabeculae from surrounding soft tissue based solely on their elevated calcium and phosphorus content [28]. This approach delineated sharp compositional boundaries at the growth plate—bone interface that were not apparent from morphology alone, demonstrating how Z-contrast imaging can resolve functional transitions within developing tissues.

**Figure 1:**
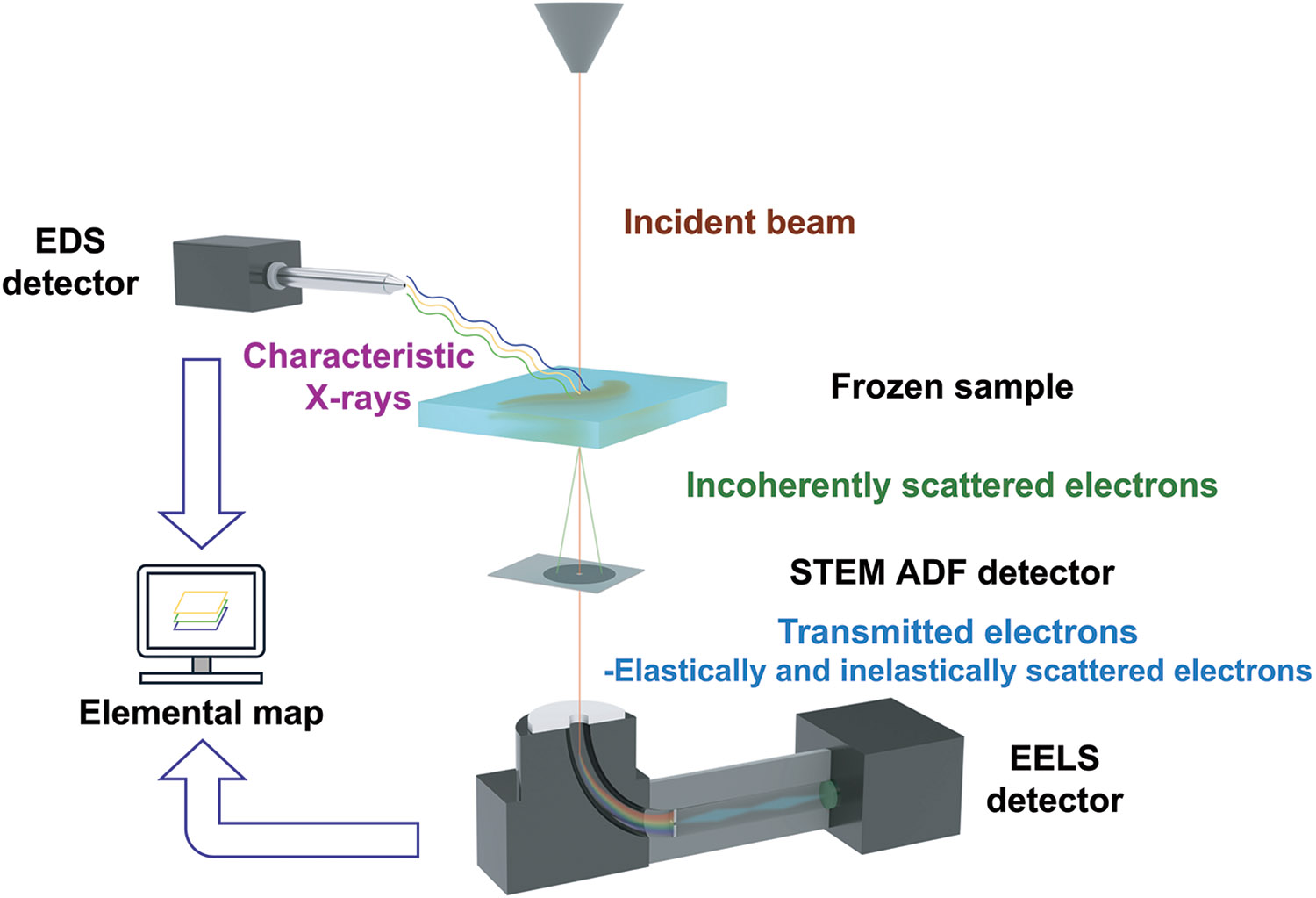
Schematic of spectroscopic imaging techniques in TEM. Incident electrons interact with the sample to produce distinct types of resultant products. Characteristic X-rays, captured by the energy dispersive spectroscopy (EDS) detector, provide element-specific fingerprints for material identification. Forward-scattered electrons, deflected at larger angles proportional to the atomic number through Rutherford scattering, are collected by an annular dark field (ADF) detector to produce Z-contrast images. Transmitted electrons are analyzed by electron energy-loss spectroscopy (EELS), which exploits intrinsic energy losses arising from electron – matter interactions to produce spectroscopic and chemical maps sensitive to elemental composition and bonding.

STEM similarly provides Z-sensitive imaging using annular dark field (ADF) detection, with the detector geometry and postspecimen lenses controlling the angular range of scattered electrons collected (Figure 1). Early cryo-STEM studies showed that dense intracellular compartments, such as polyphosphate storage granules in vitrified bacteria, can be detected by enhanced scattering alone, providing a label-free way to identify elemental enrichment [29].

Recently, 4D-STEM, also known as scanning electron nanodiffraction (SEND), has emerged as a powerful technique for the analysis of beam-sensitive materials. By acquiring a diffraction pattern at every pixel of the raster scan, this method enables the simultaneous collection of spatial and reciprocal space information. 4D-STEM also enables ptychography and tilt-corrected bright field imaging, which have been recently explored to image biological samples [30,31]. From 4D-STEM datasets, dark field images can be extracted by applying virtual detectors, whereas in conventional dark field STEM, collection angles are determined by the (fixed) camera length during acquisition [32,33]. Since this process is performed postacquisition, virtual dark field (VDF) images can be flexibly generated by segmenting specific scattering angular ranges. This flexibility allows for the precise reconstruction of Z-contrast images by optimizing the collection angles from the raw dataset. For instance, tomograms of metal nanoparticles embedded in metal—organic frameworks were successfully reconstructed via VDF imaging from low-dose 4D-STEM datasets [34]. To distinguish between the two materials, VDF detector angles were chosen based on simulations of their dominant scattering angles. While backscattered electron and dark field imaging provide a dose-efficient way to localize chemically distinct regions based on Z number, they lack elemental specificity; thus, Z-contrast imaging is often used as a simple complementary guide for subsequent spectroscopic analysis, pinpointing regions where elemental or chemical differences are likely to yield informative signals.

## Spectroscopic cryo-EM imaging: from elemental mapping to chemical states

Scanning-based imaging readily lends itself to spectroscopic interrogation of biological specimens, providing direct access to elemental composition and, in some cases, chemical state. Among the available techniques, energy-dispersive X-ray spectroscopy (EDS) and electron energy-loss spectroscopy (EELS) are the most widely applied to cryogenic, beam-sensitive biological samples, and each has distinct strengths and limitations (Figure 1). EDS identifies elements through their characteristic X-ray emission following inner-shell ionization. When high-energy incident electrons interact with the sample, inner shell electrons of an atom are ejected and generate electron vacancies. This leaves the atom in an unstable, ionized state. In order to return to a stable state, outer shell electrons transition to fill these vacancies. During this process, excess energy is emitted as a characteristic X-ray. Since each element possesses unique energy levels, these characteristic X-rays serve as a chemical fingerprint for elemental identification. While EDS is commonly used in materials science and geology, its use for biological specimens is constrained by the low signal from light elements and the high electron doses required for achieving an acceptable signal-to-noise ratio. Consequently, EDS is most effective for mapping relatively abundant or higher-Z elements (such as calcium and phosphorus), particularly in cryo-SEM or cryo-STEM workflows [35-38]. In cryogenic biological samples, EDS mapping has been used to study elemental compartmentalization. For example, cryo-SEM-EDS detected extensive phosphate accumulation within acidocalcisomes of benthic foraminifera, implicating these microbes in marine phosphorus cycling [37]. Similarly, spatially resolved EDS mapping of the human growth plate uncovered polarized calcium and phosphorus gradients correlated with mechanical stiffness and developmental function [38]. These examples highlight the capability of EDS as a powerful tool to map elemental distributions for biological and physiological roles. Nevertheless, it has inherent limitations that make it inadequate for comprehensive characterization. Due to the low fluorescence yield of light elements, low signal efficiency is a major constraint for biological samples. Consequently, utilizing EDS to map subtle concentration differences or high-resolution spatial distribution of low-Z elements is challenging. In order to compensate for the weak signals, a high electron dose is required, which can lead to severe beam-induced damage to biological samples. Moreover, while EDS can identify the presence of elements, it is impossible to distinguish their chemical states. For instance, both proteins and lipids are organic materials consisting of carbon; although EDS can confirm the existence of carbon, it cannot differentiate between peptide bonds and double bonds.

EELS complements EDS by analyzing the energy lost by electrons during inelastic scattering. When the electron beam penetrates the sample, electrons collide with atomic structures and lose specific amounts of energy. The spectrometer records this energy-loss from the transmitted electron beam. Since inner shell ionization energies are element specific, the positions of core-loss edges in the energy-loss spectrum directly identify the elements present. In addition, the near-edge fine structure encodes information about chemical bonding and the coordination environment, enabling determination of chemical states beyond elemental analysis. This EELS mechanism offers three distinct advantages that make it particularly suitable for biological analysis. First, EELS analyzes the transmitted electron beam directly, resulting in higher signal efficiency. In contrast, X-rays are emitted isotropically (4π sr), and side-entry EDS detectors collect only a small fraction of the signal due to their limited solid angle. Second, the spatial resolution of EDS is constrained by the expansion of the incident electron interaction volume due to beam broadening and secondary fluorescence. EELS can be achieved at near-atomic-level resolution by directly measuring primary inelastic scattering without secondary signal artifacts. Third, low-Z elements have low binding energies and are easily excited, resulting in EELS being exceptionally sensitive to light elements. EELS can be implemented in both conventional TEM and STEM. In TEM, widely known as energy-filtered TEM (EFTEM), the energy filter is commonly used to restrain chromatic aberration by refining the zero-loss peak. Elemental mapping is achieved using the three-window method, which utilizes two preedge windows to allow determination of the background signal, which is then subtracted from the signal in the postedge window [39]. Since EFTEM utilizes a parallel electron beam to illuminate a wide area simultaneously, it offers significant advantages in terms of dose control and rapid acquisition speed, making it particularly suitable for beam-sensitive biological samples. Utilizing this spectroscopic imaging method, Lee et al. reported LaccID as a nontoxic, genetically encodable tag to selectively visualize cell structures [40]. By generating cerium elemental maps via EFTEM, they verified that the contrast from the LaccID with Ce-DAB2 was specifically localized to the cell surface. Unabara et al. successfully visualized streptavidin-coated silica nanoparticles embedded in vitreous ice by integrating EFTEM into a conventional TEM workflow [41]. This approach allowed them to precisely distinguish the carbon-rich protein layer from silica core surrounding ice matrix.

**Figure 2:**
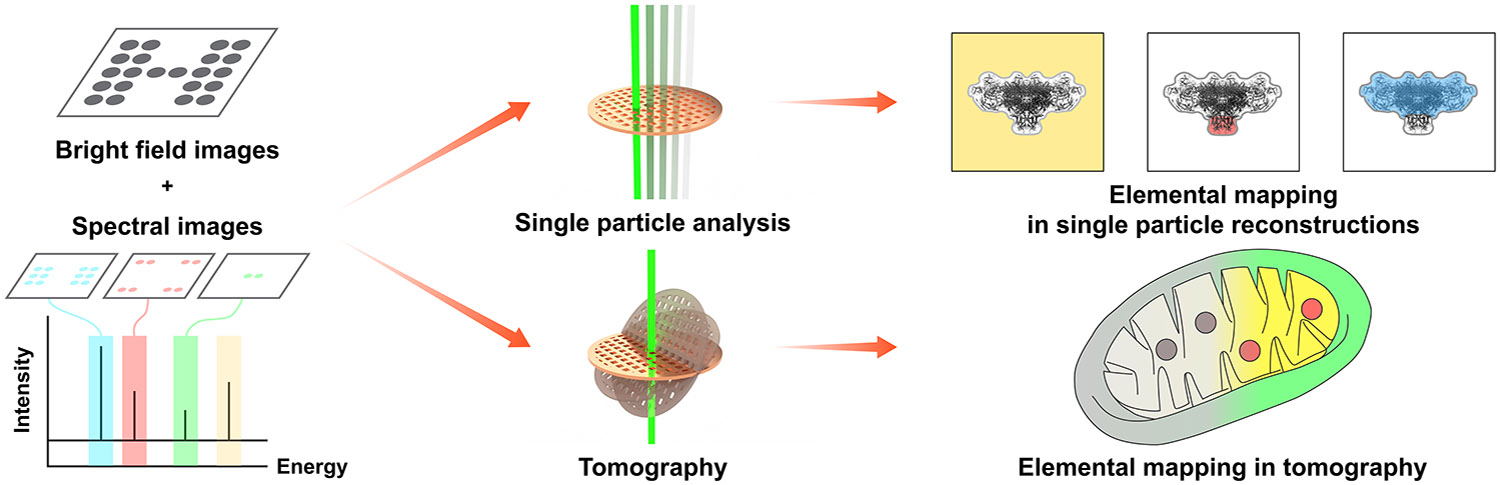
Integration of spectroscopic imaging into single-particle analysis and tomography workflows enables the mapping of chemical information onto biological structures.

Nevertheless, the three-window method is restricted to either a single or a limited number of elements per dataset. Furthermore, background modeling based on only two preedge windows is susceptible to extrapolation errors, potentially leading to inaccurate results. In contrast, STEM-EELS operates in the spectrum-imaging (SI) mode, acquiring an energy-loss spectrum for every pixel of the scan. This generates a 2D image with chemical spectrum information. Access to the full spectral range enables more sophisticated background modeling, significantly improving the accuracy of signal extraction and minimizing artifacts. Moreover, this capability allows the simultaneous extraction of information on multiple elements from a single dataset. With these distinct advantages, STEM-EELS has become a standard method, and cryo-STEM-EELS is increasingly utilized for beam-sensitive materials [42-45] and biological samples (Figure 2).

Pfeil-Gardiner et al. successfully adapted single particle analysis workflows to cryo-STEM-EELS, establishing what they termed reconstructed electron energy-loss (REEL) analysis [46]. This advance was made possible by combining an innovative data processing strategy with increased computational power and improved detector sensitivity. Because the strict dose limit (<100 e^−^/Å^2^) produces extremely weak and noisy individual EELS spectra, they decoupled the spatial alignment from the spectral signal. They utilized high-intensity low-loss images to accurately estimate particle poses, which were then used to integrate and average the noisy energy-loss spectra from multiple particles. This method achieved a sufficient signal-to-noise ratio to distinguish fine chemical features in the 3D reconstructions of rabbit RyR1 and worm hemoglobin. However, further hardware optimization will be required before REEL analysis can be used routinely. Achieving single-atom-level sensitivity requires larger accumulated datasets, and data acquisition is currently limited by detector speed. Because STEM-EELS requires a separate detector readout at each scan position, data acquisition and processing are slow, requiring several weeks of instrument time for this study. Furthermore, these slow acquisitions hinder the use of standard SPA routines like dose fraction and motion correction, as the detector cannot capture multiple frames fast enough to split the low electron dose. Integration of next-generation direct electron detectors with higher readout speeds will therefore be essential to expand dataset sizes, improve alignment accuracy, and substantially improve elemental sensitivity.

In parallel, by employing a cryo-STEM-EELS with tomography workflow, Wu et al. determined that mitochondria in optic disc drusen accumulate precipitates in the form of calcium phosphate [47]. Mapping of core-loss signals in vitrified biological samples was historically considered impossible, as the required high electron dose (>10^7^ e^−^/Å^2^) would destroy the structural context. This work was enabled by the advent of direct electron detectors. The substantial increase in detective quantum efficiency (DQE) provided by these detectors improved sensitivity to weak core-loss signals by orders of magnitude. This hardware advancement enabled the 3D elemental mapping of complex, native cellular structures within the strict dose limits of cryogenic preservation. These results highlight the potential of EELS to provide chemically specific information at nanometer resolution, capabilities fundamentally inaccessible to phase-contrast imaging.

More broadly, related developments, such as low-dose monochromated EELS, correlative electron ptychography, and applications to beam-sensitive materials or soft-matter systems, further illustrate the rapid expansion of cryogenic spectroscopic imaging beyond traditional biological targets [42-45,48,49]. Until recently, applying EELS to cryogenic biological specimens was limited not only by radiation damage but also by beam-induced charging in vitreous ice and signal loss from plural scattering [39,43]. Although advances in detector technology have improved the recovery of meaningful information under dose constraints, complementary experimental strategies remain necessary. Optimized scanning strategies, including interleaved or nonraster probe trajectories, help mitigate beam-induced charging [50,51]. Sample thickness remains a critical constraint, as plural scattering quickly obscures core-loss signals in thicker specimens [47]. Thus, cryogenic FIB milling has become essential for preparing electron-transparent lamellae from cells and tissues [7,52-55]. Beyond thinning, emerging FIB platforms that integrate time-of-flight secondary ion mass spectrometry further enable complementary correlative chemical mapping [16,38,56].

## Outlook: emerging directions for spectroscopic cryo-EM

The convergence of cryogenic preservation, advanced scanning imaging, and low-dose spectroscopy is transforming cryo-EM from a primarily structural technique into a chemically informed imaging platform. Building on the recent proof-of-principle advances discussed above, the next stage of development will depend on translating these capabilities into more scalable, targeted, and information-rich workflows for complex biological specimens. Continued improvements in hardware will remain important for broadening the applicability of spectroscopic cryo-EM under strict low-dose conditions. For EDS, larger solid-angle detector geometries will be particularly valuable because isotropic X-ray emission intrinsically limits signal collection in conventional detector configurations. By increasing the number of detected photons for a given dose and dwell time, it could enable faster acquisition and more reliable extraction of chemically meaningful information from weak signals [57]. For EELS, detector speed and sensitivity address complementary bottlenecks. Faster detectors improve the practicality of STEM-EELS by reducing the time required to acquire large spectral datasets with advances in computational real-time data processing [58,59]. More sensitive detectors, in contrast, are crucial for extracting meaningful signal under dose constraints. Also, unique detector designs may further broaden the scope of spectroscopic cryo-EM by enabling the acquisition of multimodal information within a single acquisition. For instance, hollow detector geometries could enable the simultaneous collection of EELS and 4D-STEM diffraction signals [49]. With these developments, we anticipate that the successful spectroscopic imaging strategies demonstrated in the fields of materials science and physics will be effectively adapted for biological systems [42,43,60].

Looking ahead, closer integration of Z-contrast imaging, EELS, and other correlative modalities will enable multiscale workflows in which chemically distinct regions are first identified, then selectively targeted for high-resolution analysis within the same specimen. For example, volumetric Z-contrast imaging by cryo-FIB-SEM could be used to map elemental distributions across cells or tissues, guiding subsequent cryo-EELS and/or cryo-ET interrogation of specific regions of interest. In parallel, fluorescence-guided cryo-FIB milling and cryo-lift-out approaches are increasingly enabling the selective isolation of defined cells or subcellular regions from complex tissues [8,9]. Together, these advances will extend targeted structural and chemical imaging to more heterogeneous, native contexts, substantially enriching the molecular information obtainable from a single specimen.

At the same time, advances in computational analysis are likely to improve the recovery of useful information from low-dose data. These include both more conventional strategies, such as subsampling and dictionary-learning-based inpainting [61,62], and deep-learning-based approaches for denoising, superresolution, and uncertainty-aware image restoration [63] in cryo-FIB-SEM images for biological samples. Recent AI-enabled approaches suggest several possible routes toward more efficient spectroscopic cryo-EM, including direct denoising of weak EELS signals [64], AI-guided targeted acquisition that limits spectroscopy to selected regions of interest [65] and multimodal reconstruction frameworks that combine sparse spectroscopic measurements with low-dose acquisition [66]. Although developed mainly in inorganic materials systems, these studies suggest that similar approaches will also be valuable for spectroscopic cryo-EM, particularly for recovering weak spectral signals, improving the reliability of chemical maps, and integrating sparse spectroscopic data with structural imaging.

At present, spectroscopic cryo-EM is likely to be most effective for thin, compositionally heterogeneous specimens in which specific elements or chemical states are locally enriched. With continued advances in instrumentation, multimodal workflows, and computational analysis, however, these approaches may increasingly enable discrimination of subtler compositional differences even in biological specimens dominated by light elements.

Collectively, these developments position spectroscopic cryo-EM to become an increasingly practical and powerful approach for directly linking molecular structure with chemical composition in structural biology and beyond [7,16,38,52-56].

## Data Availability

No data was used for the research described in the article.
